# Effect of neem-derived plant protection products on the isopod species *Porcellionidespruinosus* (Brandt, 1833)

**DOI:** 10.3897/zookeys.801.25510

**Published:** 2018-12-03

**Authors:** Pratik Doshi, Anett Mészárosné Pó s, Ferenc Tóth, Márk zalai, György uróczi

**Affiliations:** 1 Szent István University, Faculty of Agricultural and Environmental Sciences, Plant Protection Institute, H-2100, Páter Károly utca 1., Gödöllő, Hungary Szent Istvan University Godollo Hungary

**Keywords:** azadirachtin, biological pest control, isopod, neem leaf extract, non-target organism

## Abstract

Neem-based products have gained major attention over the last few years due to their wide range of applications in pest management, and have been in the focus of biological plant protection research in the past decade. Yet, there is limited information available to understand the side effects of these neem-derived pesticides on non-target species in soil. Therefore, *Porcellionidespruinosus*, a terrestrial isopod, was chosen as a non-target species to investigate such possible effects. Two different experiments were conducted to study two different neem-derived plant protection products, i.e., NeemAzal T/S (1% azadirachtin) which is a commercial product registered in the EU, and neem leaf extract from dried neem leaves (1%).The latter simulates the plant protection product, is domestically produced, and widely used by farmers in India and other tropical and subtropical countries. Findings are consistent with previous results obtained with other non-target organisms, i.e., neither of the tested neem products have adverse effects on the mortality of *P.pruinosus*. However, further research on a wider range of soil organisms is needed to prove the safety of neem-based products as biological control agents and to be part of integrated pest management.

## Key message

The safe use of neem (*Azadirachtaindica*)-based biological insecticides requires more information about their possible side effects on non-target organisms. Such organisms are the woodlice species, important decomposers of organic material in agricultural areas. It was demonstrated that neither a commercial product of neem nor a domestic neem leaf extract had any adverse effect on a ubiquitous woodlice species, *Porcellionidespruinosus*.

## Introduction

Different environmental conditions and chemical stressors may interact and can have a negative impact on the soil biota (Morgado et al. 2016). The long-term effects of indiscriminate and excessive use of synthetic pesticides induced research to develop alternative biological control strategies (Gurjar et al. 2012) which needs to be cost effective, non-toxic, biodegradable, eco-friendly (Girish and Bhat 2008). Such non-chemical and biological pest control methods are not only fundamental to organic farming but they are also involved in the basic principles of sustainable integrated pest management (Barzman et al. 2015). Amongst the wide variety of biological control agents available, plant-derived crude products or formulated pesticides are very popular because their use can be both efficient and economically rational (Gahukar 2014). This interest in plant derived products has brought attention to the neem tree (*Azadirachtaindica* A. Juss) which has been known in the Indian sub-continent for more than 4000 years (Boursier et al. 2011).

The neem tree, *Azadirachtaindica* is also referred to as *Meliaazadirachta* L., Indian lilac or Margosa (Koul et al. 1990). The extracts of neem tree have been studied for their diverse properties and have been used extensively against wide range of pest species (Gahukar 2014). Different biologically active compounds are found in all the parts of neem tree (Faheem et al. 2014) such as nimbin, nimbinin, meliatriol, azadirachtol, azadirone, azadirachtin, salannin, nimolicinoic acid, etc. (Koul et al. 1990) of which azadirachtin is the most widely studied compound.

Azadirachtin is a tetranortriterpenoid plant limonoid which possess anti-feedant and growth-disrupting properties. It was first isolated from seeds of *Azadirachtaindica* by Butterworth and Morgan in 1968 and its detailed structure was given by Broughton and his team in 1987 (Mordue and Blackwell 1993). Azadirachtin, the most active phytochemical component found in neem, is known to be effective against 550 insect species, many of them being major pests of cultivated crops (Mondal and Mondal 2012). Due to worldwide demand of organic cotton, azadirachtin-based insecticides have been gaining popularity in plant protection of this plant (Gahukar 2000).

Neem extracts and products have been used against different orders of insects such as Coleoptera (for example Jilani and Saxena (1990), Reed et al. (1982), Zehnder and Warthen (1988)) and Hemiptera (for example Abudulai et al. (2003), dos Santos-Cividanes et al. (2004)). Klemm and Schmutterer (1993) studied the effects of neem preparation on *Plutellaxylostella* (Linnaeus) (Lepidoptera), one of the most important cabbage pests in agriculture. Extensive research is being conducted with neem and its derived products against target pests. Abudulai et al. (2003) found that a commercial product of neem had an antifeedant effect as well, as it affected development and molting. Dos Santos-Cividanes et al. (2004) conducted an experiment to check the effect of neem seed powder on *Aphisgossypii* (Glover) and found that the aqueous neem seed extract is efficient in nymph mortality and reducing the survival period and fecundity of the cotton aphid.

The effect of neem-derived products on non-target organisms has also been studied. However, information on the effect of neem-based pesticides and formulations on non-target organisms of soil biota are still limited. Scott and Kaushik (1998) found that neem products have a threshold chronic toxicity on non-target organisms like the crustaceans *Daphniamagna* (Straus, 1820) and *Hyalellaazteca* (Saussure, 1858). Wagenhoff et al. (2013) tested the effects of NeemAzal T/S on reproductive output of *Nicrophorusvespilloides* (Herbst 1783), a common burying beetle, feeding on a variety of animal carcasses. *Nicrophorusvespilloides* were fed with cockchafers which were previously fed with neem-treated leaves. They found that it had no negative impact on the reproduction nor they found any impacts on the morphology of *N.vespilloides*.

Woodlice species (Isopoda, Oniscidae) are ubiquitous saprophagous members of the soil fauna (Paoletti and Hassall 1999). They are present in various densities both in conventional and organic farming systems (ibid), and as such exposed to any pesticide treatment. Isopods can also be used for biomonitoring, both in contaminated or remediated areas (Loureiro et al. 2006). Isopods inhabit littoral zone, beach, grassland, woodland, desert, and more special habitats (Warburg 1987). Adaptations to these environments are thought to be largely behavioral but it now appears that there are also well-established physiological adaptations, based on anatomical structures. Certain terrestrial isopod genera are able to detect chemical cues using their second antenna pair (Harzsch et al. 2011). This can explain the results of Santos et al. (2011) where the binary combinations of dimethoate, glyphosate and spirodiclofen, an insecticide and an herbicide and an acaricide respectively, resulted a dose related avoidance response of *P.pruinosus*.

In another study, single and combined toxicity of atrazine, dimethoate, lindane, zinc and cadmium were tested in *Porcellionidespruinosus* (Brandt, 1833) and *Enchytraeusalbidus* (Henle, 1837) an annelid, commonly known as white worm, using avoidance as test parameter. For both the species, patterns of antagonism were found when exposed to dimethoate and atrazine, synergism for lindane, dimethoate, and atrazine, synergism for lindane and dimethoate exposures and concentration addition for cadmium and zinc occurred, while the exposure to cadmium and dimethoate showed dissimilar patterns (Loureiro et al. 2009).

This soil ’cleaning’ result can be defined as a positive ecosystem service (ES) (MA 2005). ES can provide numerous goods and services by the organisms, guilds, and ecological communities to society (Garbach et al. 2014). Soil functions are strictly dependent on structure and biodiversity. They exposed to several physical, chemical, and biological stressors, which are directly or indirectly related to anthropogenic activities (Morgado et al. 2018). If the biodiversity is not too affected by these it can provide other important roles like decomposition (Jia et al. 2015; El-Wakeil 2015), or heavy metal accumulation (Mazzei et al. 2014) that especially isopods execute. Moreover just with their presence the biodiversity becomes more complete and more balanced (Pokarzhevskii et al. 2003; Smeding and de Snoo 2003).

A small-scale terrestrial ecosystem containing soil collected from an agricultural field in Central Portugal was used to evaluate the effects of the combination of the herbicide glyphosate and the insecticide dimethoate in another study. The application of dimethoate led to a decrease in feeding activity in all concentrations tested. The mortality of isopods exposed to dimethoate in single and binary exposures was high. Exposure to dimethoate decreased the acetylcholinesterase activity of isopods (Santos et al. 2011).

In this paper we present results on the side-effect of NeemAzal T/S and neem leaf extract on the terrestrial isopod species *Porcellionidespruinosus*. We selected *P.pruinosus* as a non-target organism, because it is ubiquitous and it occurs in anthropogenic environments, where pest control is applied. They play a vital role in the fragmentation and decomposition process of leaf litter, thereby causing changes in soil quality and soil services (Ferreira et al. 2016).

## Materials and methods

The methodology of Akca et al. (2015) was followed with modifications. Experiments were carried out with six different concentrations of both NeemAzal T/S and neem leaf extract with a control, each replicated ten times under laboratory conditions.

*Collection of isopod species*: *Porcellionidespruinosus* adults were collected from Regional Waste Management Center Pusztazámor, Hungary, by hand sorting. Isopods were bred and maintained at the Institute of Plant Protection of Szent István University, Gödöllő, Hungary. Species level identification was based on the taxonomic key developed by Brandt (1833) (Farkas and Vilisics 2013).

*Preparation of neem leaf extract*: For neem leaf extract, air-dried neem leaves were obtained from local growers in India, Maharashtra, Konkan Division, Mumbai Suburban area. A stock concentration of 1% was prepared by soaking 1g of crushed dried neem leaves in 100 ml distilled water overnight and then filtered using a non-sterile filter paper. Different working concentrations (0.05, 0.1, 0.25, 0.5, 0.75, and 1%) of neem leaf extract were prepared from 1% stock solution using distilled water in the laboratory and were used on the same day. Generally the applied dosage used by local growers in India has a maximum concentration of 1%. In this experiment, we tried to model the concentration used by the local growers in the field conditions.

*Preparation of azadirachtin*: NeemAzal T/S (Trifolio-M GmBH), a commercial product containing 1% azadirachtin, registered in the EU, was used. A stock concentration of 1% azadirachtin was prepared (from NeemAzal T/S which is 1% azadirachtin) by diluting 1 ml NeemAzal T/S in 100 ml of distilled water which equals to 0.01% azadirachtin. It was further diluted to get the 0.0005, 0.001, 0.0025, 0.005, 0.0075 and 0.01% azadirachtin concentrations respectively and was used on the same day. The registered dosage of azadirachtin ranges from 0.0025 to 0.005%, depending on the plant culture in the EU.

A control with only distilled water was used for both experiments. The working concentrations and distilled water were sprayed using a hand sprayer under laboratory conditions.

*Experimentaldesign*: Five adults of *Porcellionidespruinosus* were placed in glass Petri dishes (13 cm in diameter), with 1 g of commercial horticultural soil (pH = 7.0) and approximately 1 g of fresh potato as a food source. Two milliliters of different working concentrations of neem leaf extract and azadirachtin were sprayed using a hand sprayer. After spraying, the Petri dishes were kept in the dark, checked after time periods of 1, 24, 48, 72, 96, and 120 hours post-application of neem leaf extract and azadirachtin respectively, and mortality data was recorded. The mortality data obtained after 120 hrs was subjected to statistical analysis using R software (R Core Team 2017). Logistic regressions were fitted (as the response was binary, i.e., the isopods were either dead or alive) to check the effect of the two different products on isopod mortality. To test whether the concentrations have significant effect on mortality, chi-squared tests were performed on model deviances. Prior to running the tests model diagnostic plots were investigated to assess homoscedasticity and residual normality (Faraway 2002).

## Results

The mortality of *P.pruinosus* was generally low in all treatments. In case of azadirachtin, even after 120 hours zero mortality was observed in seven replicates of 0.0005% concentration, eight replicates at 0.001%, nine replicates of 0.0025%, seven replicates of 0.005 and 0.0075% each, and four replicates of 0.01%.

The same was observed in the case of neem leaf extract, after the time period of 120 hours: zero mortality in case of five replicates of 0.05% concentration, nine replicates of 0.1%, six replicates of 0.25%, seven of 0.5%, four replicates of 0.75%, and five replicates of 1% (see Table 2).

The mortality slightly increased with the concentration but this observed increment was not statistically significant (Table 2). Unusually high values (i.e., higher mortality) were occasionally observed both in NeemAzal T/S and neem-leaf extract treatments. These can be attributed either to the juvenile mortality of *P.pruinosus* (Dangerfield and Telford 1995) or suboptimal conditions.

**Figure 1.**
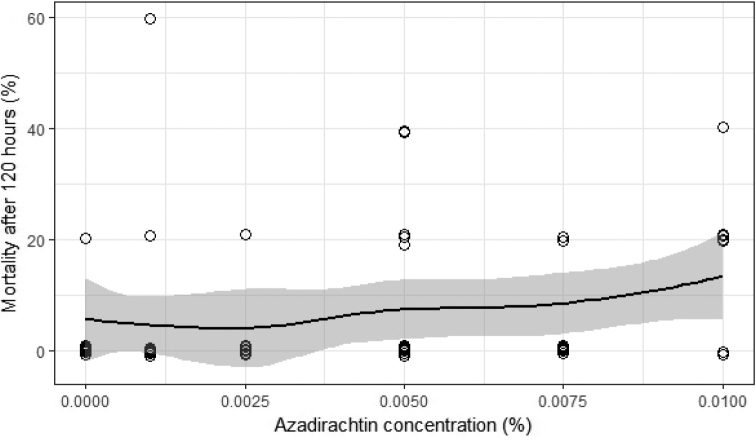
Mortality rate of the isopod *Porcellionidespruinosus* after 120 hours at different concentrations of NeemAzal T/S (1% azadirachtin). The vertically jittered circles (to avoid perfect overlapping) indicate the individual isopods whereas the line indicates the trend of the mortality with respect to increasing concentrations and the grey area represents the 95% confidence level.

**Figure 2.**
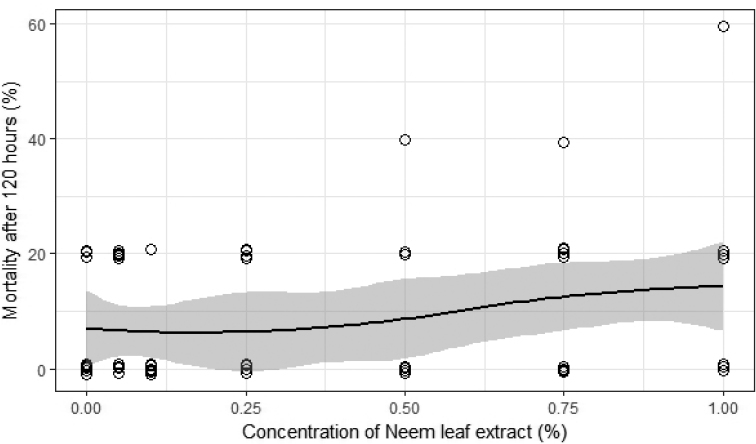
Mortality rate of the isopod *Porcellionidespruinosus* after 120 hours to at different concentrations of neem leaf extract. The vertically jittered circles (to avoid perfect overlapping) indicate the individual isopods whereas the line indicates the trend of the mortality with respect to increasing concentrations and the grey area being the 95% confidence level.

Different concentrations of NeemAzal T/S and neem leaf extracts were compared to check their respective effects on the mortality of the isopods. Neither azadirachtin nor neem leaf extract affected the observed isopod mortality (p-values are 0.43 and 0.39 and McFadden’s pseudo R^2^: 0.04 for azadirachtin, 0.05 for neem leaf extract respectively; Figs 1, 2).

**Table 1.** Effect of NeemAzal on the mortality of *Porcellionidespruinosus* expressed as cumulative mean for different time intervals.

Treatment	conc (%)		Mean mortality rate after time interval					
			1 hr	24 hrs	48 hrs	72 hrs	96 hrs	120 hrs
azadirachtin (NeemAzal T/S)	0	mean	0.1	0.1	0.1	0.1	0.1	0.2
		SD	0.32	0.32	0.32	0.32	0.32	0.42
	0.0005	mean	0.2	0.3	0.3	0.3	0.3	0.5
		SD	0.42	0.67	0.67	0.67	0.67	0.85
	0.001	mean	0	0.3	0.3	0.3	0.4	0.4
		SD	0	0.95	0.95	0.95	0.97	0.97
	0.0025	mean	0	0	0	0	0	0.1
		SD	0	0	0	0	0	0.32
	0.005	mean	0.1	0.2	0.2	0.2	0.2	0.3
		SD	0.32	0.42	0.42	0.42	0.42	0.48
	0.0075	mean	0.2	0.2	0.2	0.2	0.2	0.3
		SD	0.42	0.42	0.42	0.42	0.42	0.48
	0.01	mean	0	0.1	0.5	0.5	0.5	0.7
		SD	0	0.42	0.71	0.71	0.71	0.67

Key: conc = concentration, SD= Standard deviation. Each value is an average of ten replicates.

**Table 2.** Effect of neem leaf extract on the mortality of *Porcellionidespruinosus* expressed as cumulative mean for different time intervals.

**Treatment**	**conc (%)**		**Mean mortality rate after time interval**					
			**1 hr**	**24 hrs**	**48 hrs**	**72 hrs**	**96 hrs**	**120 hrs**
neem leaf extract	0	mean	0	0	0	0	0.1	0.33
		SD	0	0	0	0	0.33	0.5
	0.05	mean	0	0	0	0.1	0.2	0.527
		SD	0	0	0	0.32	0.42	0.52
	0.1	mean	0	0	0	0	0	0.1
		SD	0	0	0	0	0	0.32
	0.25	mean	0	0	0	0	0.2	0.4
		SD	0	0	0	0	0.42	0.52
	0.5	mean	0	0	0	0	0	0.4
		SD	0	0	0	0	0	0.7
	0.75	mean	0	0.1	0.2	0.2	0.2	0.7
		SD	0	0.32	0.42	0.42	0.42	0.67
	1	mean	0	0.1	0.2	0.3	0.4	0.7
		SD	0	0.32	0.42	0.67	0.7	0.95

Key: conc = concentration, SD= Standard deviation. Each value is an average of ten replicates.

## Discussion

While there are numerous literature references available on the effect of neem and neem-derived products on target organisms, some of the studies reported data on non-target organisms as well. For instance, Goktepe et al. (2004) carried out an ecological risk assessment of neem-based products on six aquatic animals through short-term acute toxicity tests and concluded that the risk values did not exceed the criteria and were safe for use. In contrast, it has been noted that neem components do have adverse effects on non-target aquatic organisms such as *Daphnia* species (Straus, 1820) as studied by Stark (1997) and fish (Tangtong and Wattanasirmkit 1997). Scott and Kaushik (1998) assessed the effect of Margosan-O (a product of neem seeds) on non-target aquatic invertebrates. Their investigation revealed that there can be some effects of the product on non-target organisms at higher concentration but if applied in agricultural systems, Margosan-O may not reduce the survival or reproduction of the non-target aquatic organisms. Wagenhoff et al. (2013) studied the effects of NeemAzal T/S on the burying beetle *Nicrophorusvespilloides*, which co-occurs with the forest cockchafer *Melolonthahippocastani* (Fabricius, 1801) and also feeds on the carcasses of *M.hippocastani*. In their study, they fed *N.vespilloides* with dead *M.hippocastani* which were previously fed with neem-treated leaves. They neither observed any impact on the mean larval weights nor on the morphology of *N.vespilloides*. Still, they authors did not dismiss the possibility of azadirachtin passing through the food chain and affecting other non-target organisms.

Akca et al. (2015) investigated the effect of azadirachtin (NeemAzal T/S) on terrestrial isopod *Philosciamuscorum* (Scopoli, 1763) and did not find any negative effects on *P.muscorum*. The results of our experiments were found to be similar and this experiment for the first time investigated the effects of two different neem products on this non-target isopod species, i.e., *Porcellionidespruinosus*.

From our results it can be concluded that neither NeemAzal T/S nor neem leaf extracts pose any risk to the terrestrial isopod species studied in the tested concentrations. However, further research is needed to test the possible effect of various neem products on the members of the soil fauna. Also, it can be concluded that NeemAzal T/S and domestic neem leaf extract do not differ in respect to their mortality effects on *P.pruinosus*.

## Author contribution statement

PD and AMP designed and conducted the experiments. MS analyzed the data. PD wrote the manuscript. AMP, FT, GT, and MS gave their feedback. All authors read and approved the manuscript.
